# External Validation of the Toulouse-Rangueil Predictive Model to Estimate Donor Renal Function After Living Donor Nephrectomy

**DOI:** 10.3389/ti.2023.11151

**Published:** 2023-03-17

**Authors:** Manuela Almeida, Gonçalo Calheiros Cruz, Círia Sousa, Cátia Figueiredo, Sofia Ventura, José Silvano, Sofia Pedroso, La Salete Martins, Miguel Ramos, Jorge Malheiro

**Affiliations:** ^1^ Nephrology Department, Centro Hospitalar Universitário do Porto, Porto, Portugal; ^2^ Unit for Multidisciplinary Research in Biomedicine, Abel Salazar Institute of Biomedical Sciences, University of Porto, Porto, Portugal; ^3^ Hospital Garcia de Orta, Almada, Portugal; ^4^ Centro Hospitalar de Trás os Montes e Alto Douro, Vila Real, Portugal; ^5^ Centro Hospitalar do Médio Tejo, Tomar, Portugal; ^6^ Departamento de Cirurgia, Centro Hospitalar Universitário do Porto, Porto, Portugal

**Keywords:** external validation, predictive model, living donor renal function, kidney transplantation, chronic kidney disease

## Abstract

A predictive model to estimate post-donation glomerular filtration rate (eGFR) and risk of CKD at 1-year was developed from a Toulouse-Rangueil cohort in 2017 and showed an excellent correlation to the observed 1-year post-donation eGFR. We retrospectively analyzed all living donor kidney transplants performed at a single center from 1998 to 2020. Observed eGFR using CKD-EPI formula at 1-year post-donation was compared to the predicted eGFR using the formula eGFR (CKD-EPI, mL/min/1.73 m^2^) = 31.71+ (0.521 × preoperative eGFR) − (0.314 × age). 333 donors were evaluated. A good correlation (Pearson *r* = 0.67; *p* < 0.001) and concordance (Bland-Altman plot with 95% limits of agreement −21.41–26.47 mL/min/1.73 m^2^; *p* < 0.001) between predicted and observed 1-year post-donation eGFR were observed. The area under the ROC curve showed a good discriminative ability of the formula in predicting observed CKD at 1-year post-donation (AUC = 0.83; 95% CI: 0.78–0.88; *p* < 0.001) with optimal cutoff corresponding to a predicted eGFR of 65.25 mL/min/1.73 m^2^ in which the sensibility and specificity to predict CKD were respectively 77% and 75%. The model was successfully validated in our cohort, a different European population. It represents a simple and accurate tool to assist in evaluating potential donors.

## Graphical Abstract

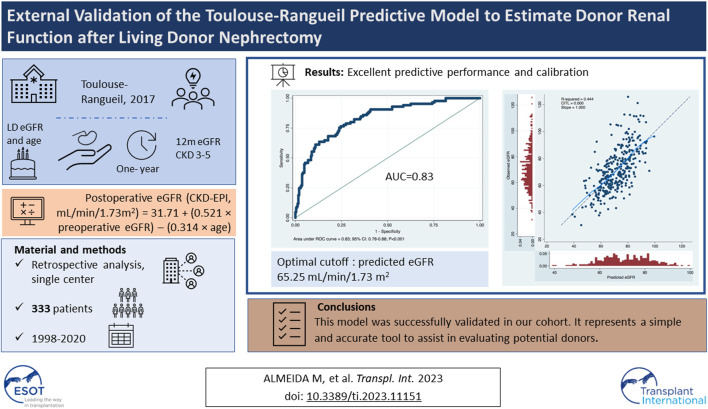

## Introduction

Living donor kidney transplant is the best treatment for ESRD patients eligible for transplant (1, 2). Living donation increases organ availability, decreases time on the waiting list, allows pre-emptive transplantation, and improves graft and patient survival (1–3).

The evaluation of a living donor candidate is a multidisciplinary task to minimize the risk for the donor while ensuring the organ’s suitability for the recipient (4, 5). Despite being the only surgical indication that grants no direct medical benefit to a healthy patient, a living nephrectomy is considered a safe procedure for the donor (5–7). Long-term follow-up data, however, have shown that donors are at an increased risk of CKD and, rarely, ESRD compared to healthy non-donors (6–9) As such, these patients would be subjected to the cardiovascular and global morbidity and mortality of CKD (10). Furthermore, the increasing acceptance of donors with increasing age or with minor medical changes that were previously declined (6), makes the issue of kidney donors’ safety of utmost importance (6). Moreover, the scarcity of good-quality studies on their long-term follow-up must be acknowledged (6, 7).

Current Clinical practice guidelines on the evaluation and care of living kidney donors from Kidney Disease Improving Global Outcomes (KDIGO) recommend a comprehensive approach to risk assessment that should replace decisions based on assessments of single risk factors evaluation (4). Transplant programs should provide each donor candidate with individualized quantitative risks from donation and communicate them clearly to donor candidates (4). Furthermore, each donor candidate’s risk should be compared to predetermined thresholds for acceptance and declined if the risk exceeds the acceptable limit for the Transplant Unit (4). Nevertheless, precise tools to quantify individualized donor risks are lacking.

A predictive model to estimate the donor 1-year post-donation estimated glomerular filtration rate (eGFR) and risk of CKD was developed from a Toulouse-Rangueil cohort in 2017 (11). Benoit et al. retrospectively evaluated a single-center French cohort of 202 living donors and identified age and preoperative eGFR as independent predictors of postoperative eGFR. A formula using multiple linear regression was designed for clinical application and the authors described a good statistical performance (11). This model was then externally validated in a German center by Kullik et al. (12) and in a different French cohort (13) and was shown to have a good correlation to the observed 1-year post-donation eGFR.

We sought to externally validate this predictive tool in a different, large European cohort of patients who underwent a living donor kidney transplant at our center.

## Material and Methods

This external validation study was conducted according to the Transparent Reporting of a multivariable prediction model for Individual Prognosis Or Diagnostics (TRIPOD) guidelines (14).

We retrospectively reviewed the clinical data of all (*n* = 366) the donors who underwent nephrectomy for living donor kidney transplantation at our institution between 1998 and December 2019. After excluding 33 donors, in whom eGFR at 1 year was missing, the remaining 333 donors were included in this study.

Following international guidelines, all donors were subjected to a standard evaluation protocol. Baseline demographic, anthropomorphic, analytical, and clinical data were collected from the living kidney donors. Serum creatinine Serum creatinine-based CKD-EPI equation (15) was used to predict eGFR. Split renal function was evaluated by Nuclear Renography and renal anatomy by a Computed Tomography scan.

Hypertension was defined by blood pressure in the consultation >140/90 mmHg, ABPM > 135/85 mmHg, and past diagnosis of hypertension or antihypertensive medication. Uncontrolled hypertension or evidence of end-organ damage were criteria of exclusion. Potential donors with a history of malignancy, obesity, or diabetes were excluded. Although a lower limit of eGFR was not established by Unit protocol, potential donors with eGFR below 80 m mL/min/1.73 m^2^ were usually discarded. The final approval for kidney donation was reviewed in a multidisciplinary meeting and the ethical approval was mandatory.

Left-side procurement was preferred for anatomical reasons except for complex vessels anatomy or when a significant renal asymmetry was found, and the right kidney had the lower clearance. A transperitoneal laparoscopic approach was performed in most donors. Lifetime annual follow-up appointments are available for all donors.

For validation of the predictive model, eGFR was calculated using the CKD-EPI Chronic Kidney Disease Epidemiology pre-donation and 1 year (±30 days) after donation.

### Statistical Analysis

Data are presented as mean (and standard deviations for continuous variables and frequency (and percentages) for categorical variables.

Observed eGFR using CKD-EPI formula at 1-year post-donation was compared to the predicted eGFR using the formula developed in Toulouse-Rangueil: postoperative eGFR (CKD-EPI, mL/min/1.73 m^2^) = 31.71 + (0.521 × preoperative eGFR) − (0.314 × age).

The ability of this formula to predict the observed GFR was analyzed by Pearson correlation, and agreement was explored by the Bland-Altman plot. The discriminative ability to predict CKD3-5 was evaluated by the area under the receiver operating characteristic (ROC) curve and using sensitivity, specificity, and positive, or negative predictive values (PPV or NPV). Furthermore, the accuracy of the predictive model was depicted by constructing a calibration plot and assessed through the calibration slope and the calibration in the large.

A 2-sided *p*-value < 0.05 was considered as statistically significant. Statistical calculations were performed using STATA/MP, version 15.1 (Stata Corp, College Station, TX, United States).

## Results

### Baseline Characteristics

The baseline donors’ characteristics for the cohort of 333 patients are presented in Table 1. The mean donor age was 47.3 ± 10.6 years old (age range 20.7–76.2 years old), and most were female (71%). The mean body mass index was 25.3 ± 3.4 Kg/m^2^. Fifty donors (15%) were hypertensive pre-donation, and fifty-one (15%) had smoking habits. Pre-donation mean eGFR was 100.3 ± 14.7 mL/min/1.73 m^2^, while the mean 1-year post-donation eGFR was 71.4 ± 16.2 mL/min/1.73 m^2^. The mean predicted 1-year post-donation GFR was 69.1 ± 10.0 mL/min/1.73 m^2^.

**TABLE 1:** Patients’ characteristics of the 333 living donors.

	N = 333
Age, mean ± SD	47.3 ± 10.6
Sex F:M, n (%)	236 (71):97 (29)
BMI, mean ± SD (Kg/m^2^)	25.3 ± 3.4
Smoking habits, n (%)	51 (15)
Hypertension, n (%)	50 (15)
Pre-donation SCr, mean ± SD (mg/dL)	0.75 ± 0.16
Pre- donation eGFR, mean ± SD	100.3 ± 14.7
1-year postdonation SCr, mean ± SD (mg/dL)	1.05 ± 0.23
1-year postdonation eGFR, mean ± SD	71.4 ± 16.2
Predicted 1-year postdonation eGFR, mean ± SD	69.1 ± 10.0

eGFR: mL/min/1.73 m^2^.

Eighty-five donors (25.5%) reached the definition of CKD at 1-year after donation as depicted in Table 2.

**TABLE 2:** ROC: McNemar's exact test for optimal cutoff and for CKD cutoff.

		Observed eGFR		Total
		<60	≥60	
Predicted eGFR	<65.25	65 (76)	61 (25)	126
	≥65.25	20 (24)	187 (75)	207
Total		85	248	333
McNemar’s exact test *p* < 0.001, Sensitivity 77%, Specificity 75%, PPV 52%, NPV 90%				
Predicted eGFR	<60	40 (47)	17 (7)	57
	≥60	45 (53)	231 (93)	276
Total		85	248	333
McNemar’s exact test *p* < 0.001, Sensitivity 47%, Specificity 93%, PPV 70%, NPV 84%				

eGFR: mL/min/1.73 m^2^.

**FIGURE 1:**
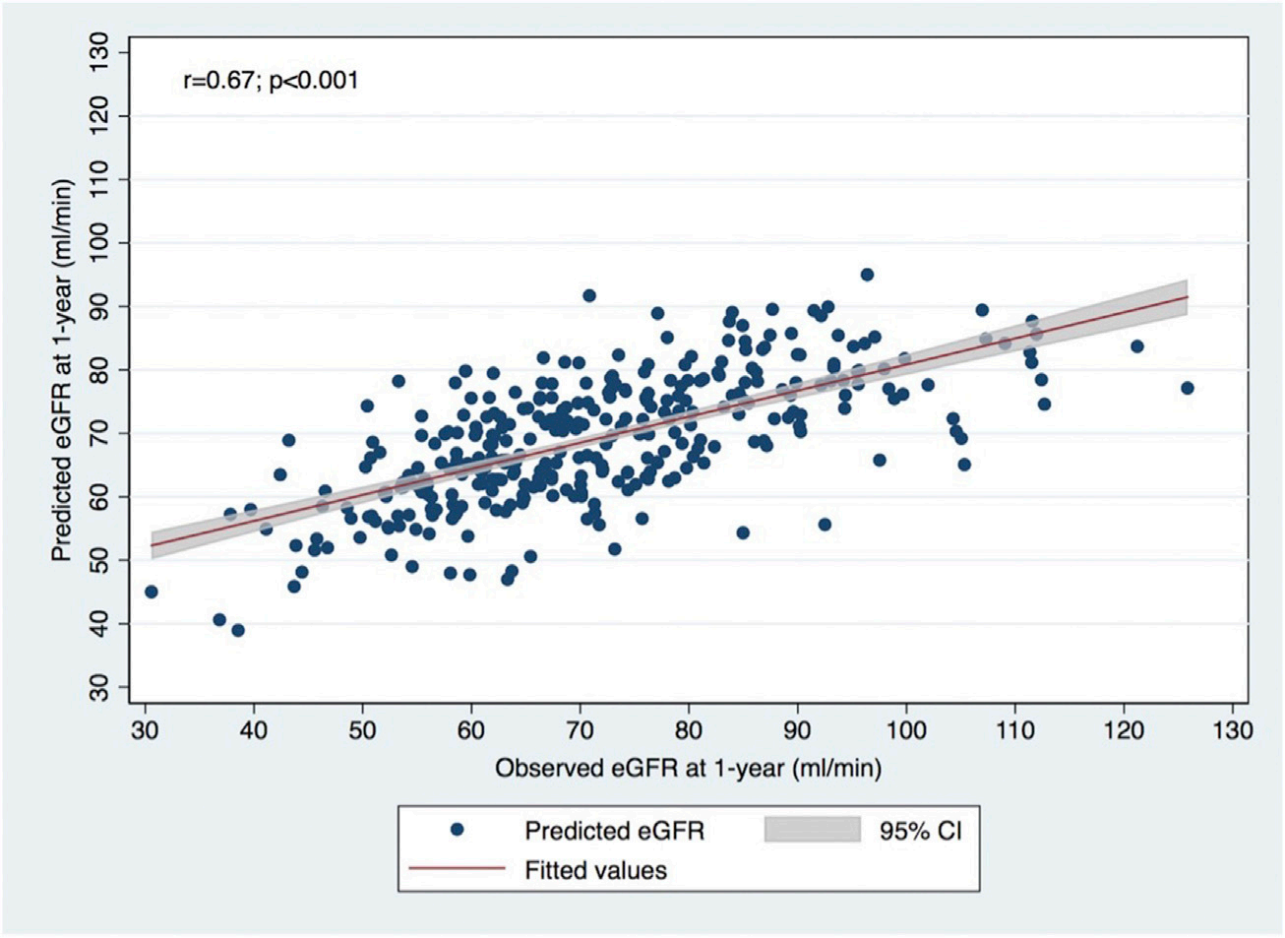
Correlation between observed eGFR using CKD-EPI formula at 1-year post-donation and predicted eGFR using the formula developed in Toulouse-Rangueil.

**FIGURE 2:**
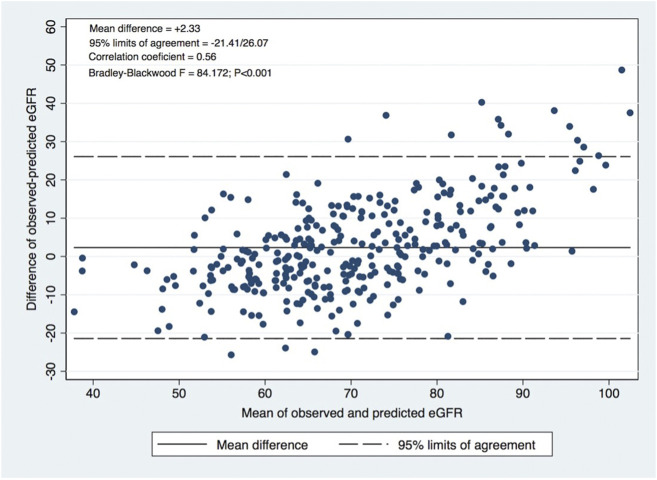
Bland-Altman plot: Agreement evaluation, correlation coefficient between the difference and the mean of observed and predicted eGFR.

A significant correlation was observed between calculated and observed 1-year eGFR (*p* < 0.001; Pearson *R* = 0.67), as shown in Figure 1. The concordance is represented by the Bland-Altman plot with a mean difference of observed-predicted eGFR = +2.33 mL/min/1.73 m^2^ (95% limits of agreement −21.41–26.47 mL/min/1.73 m^2^; *p* < 0.001) (Figure 2).

**FIGURE 3:**
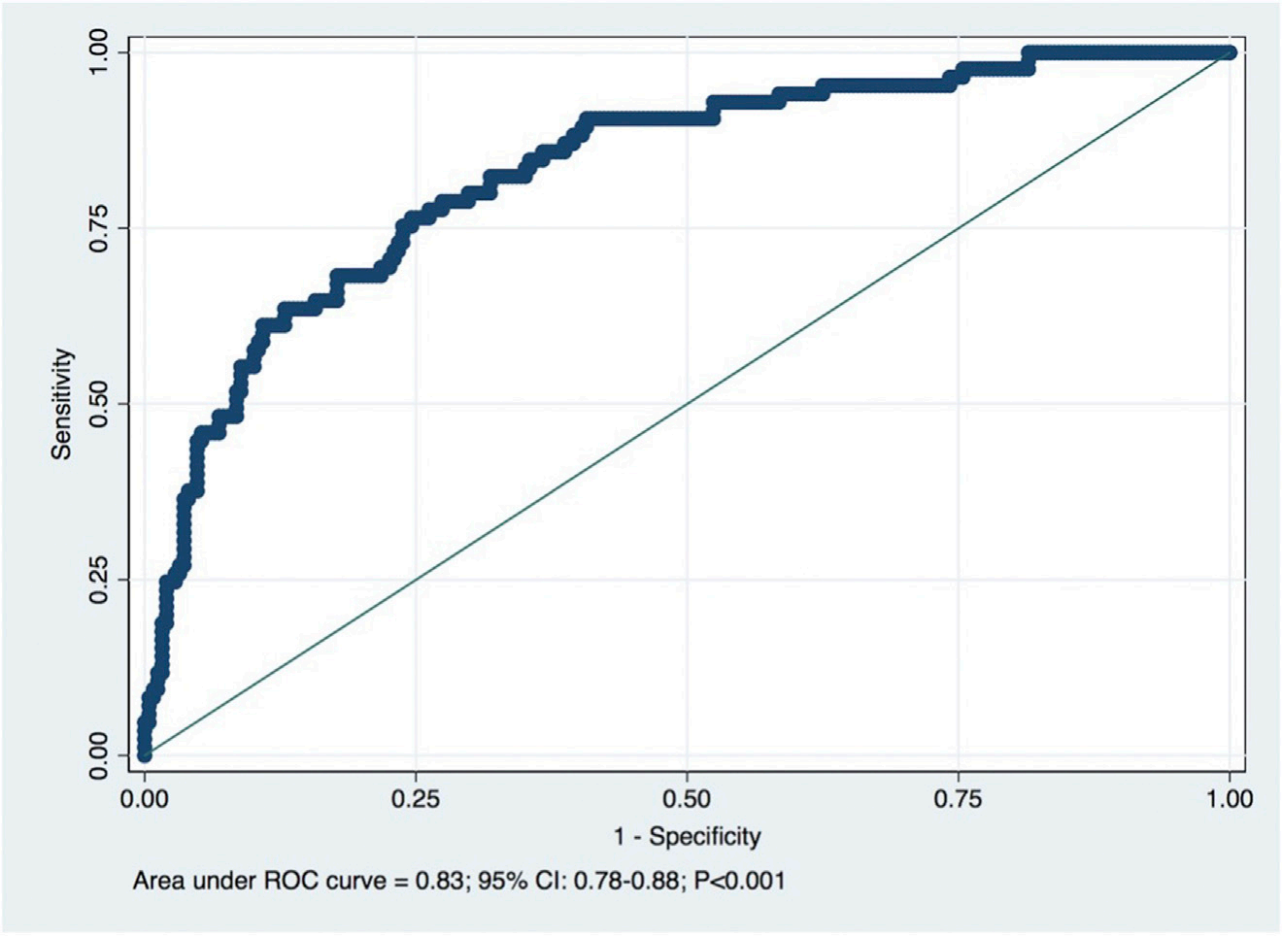
Receiver operating characteristic (ROC) curve for predicted eGFR for the detection of CKD (eGFR < 60 mL/min/1.73 m^2^). Diagonal line is the reference line: AUC = 0.83. Optimal cutoff: 65.25 mL/min/1.73 m^2^.

Furthermore, the model showed a good discriminative ability of the formula in predicting observed CKD at 1-year post-donation, with the area under the receiver operating characteristic (ROC) curve of 0.83 (95% CI: 0.78–0.88; *p* < 0.001), as shown in Figure 3, with optimal cutoff (by Youden criteria) corresponding to a predicted eGFR of 65.25 mL/min/1.73 m^2^ (5.25 mL above the equality cutoff), for which the sensibility and specificity to predict CKD were respectively 77% and 75% (Table 2). Overall, the model performance was similar in females and males (data not shown), although the optimal cutoff for the female sex corresponded to 62.23 mL/min/1.73 m^2^ (2.23 mL above the equality cutoff), for which the sensibility and specificity to predict CKD were respectively 66% and 85%. For the male sex, the optimal cutoff was similar to the global cohort, for which the sensibility and specificity to predict CKD were 77% and 82%, respectively.

**FIGURE 4:**
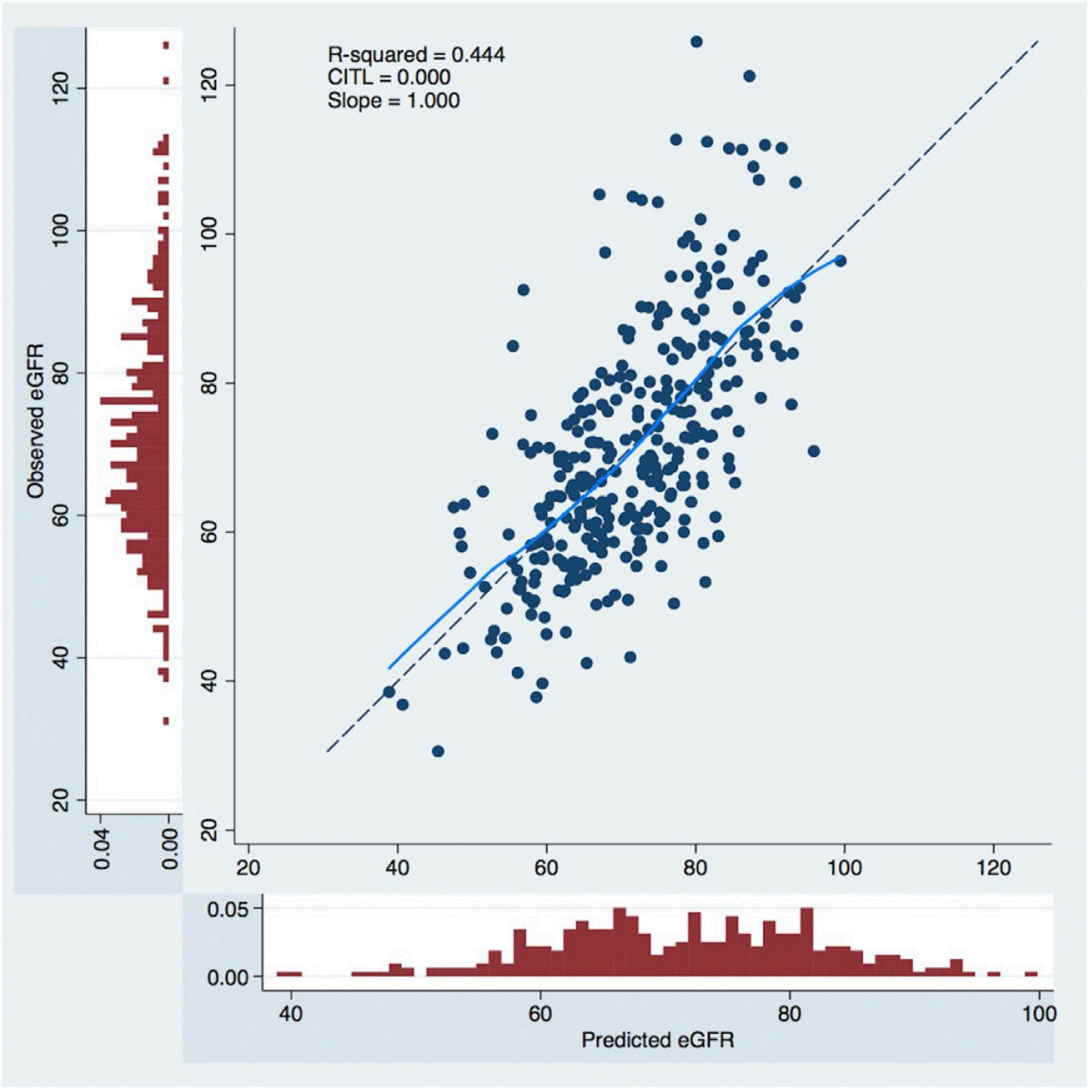
Calibration curves to predict 1-year postoperative eGFR. The *x*-axis represents model predictions, the *y*-axis the observed eGFR at 1-year. CITL, calibration in the large; eGFR, estimated glomerular filtration rate, mL/min/1.73.

The Calibration curves illustrated the model’s accuracy in the prediction of eGFR <60 mL/min/1.73 m^2^ at 1 year. The calibration curve, shown in Figure 4, exhibited an excellent prediction with a slope = 1.000 and a Calibration In The Large (CITL) = 0.000.

## Discussion

In this study, the predictive model developed at Toulouse-Rangueil (11) was validated externally in our cohort of living kidney donors in concordance with other external validation studies in different European populations (12, 13). A significant correlation was observed between calculated and observed 1-year eGFR (Pearson *r* = 0.67), and for the prediction of CKD (eGFR values < 60 mL/min/1.73 m^2^) at 1 year after donation, the model presented an AUROC of 0.83, which represents an excellent performance. Benoit et al. (13), in a population of 400 French living donors that performed nephrectomy at Necker Hospital, also described a significant correlation between predicted and observed 1-year eGFR (Pearson *r* = 0.66), and for the prediction of CKD at 1 year, the model presented an AUROC of 0.86. We must emphasize that the optimal value of predicted eGFR was around 5 mL/min higher than the equality cutoff for CKD detection at 1 year, an outcome that was correctly predicted (both its presence and absence) in every 3 out of 4 donors. This tool represents a non-invasive, low cost and readily available tool that can be joined to the living donor evaluation routine consultation, improving the living donor risk estimation and the informed consent process. The predicted eGFR value ≥ 65.25 mL/min was associated with a very high NPV (90%), identifying donors that are clearly admissible concerning renal function (Table 2). Otherwise, a predicted eGFR < 60 mL/min was associated with a high PPV (70%), identifying donors that probably should not be accepted, concerning their renal function. Anyway, a global risk assessment is mandatory (4). An older donor will have a lower 1-year eGFR, and the lower expected lifespan will mitigate a higher chance of CKD, but the expected risk of ESRD compared to a younger donor.

LDKT is considered safe, but some donors will develop CKD. And, rarely, ESRD. Two landmark studies in the living kidney donation (8, 9) made this discussion more pertinent. Furthermore, the characteristics of our living donors are changing. We are facing a population increasingly older in dialysis, and their potential donors are also older, with an increasing chance of having borderline preoperative eGFR. In this tool, age, and preoperative CKD-EPI eGFR were shown to be independent predictors of 1-year postoperative renal function.

The evaluation of the glomerular filtration rate is a crucial point in LKD. We used eGFR based on serum creatinine determinations because it is feasible and is the most common method worldwide (4). More reliable methods of isotopic evaluation are not routinely available (4). In a large retrospective study, Stevens et al. (16) demonstrated that CKD-EPI estimates were more accurate than MDRD estimates considering the actual GFR measured by urinary or plasma clearance of exogenous filtration markers. It suggests that the CKD-EPI method must be preferred in the clinical practice (16). Most transplant centers use CKD-EPI equation eGFR in the initial assessment of renal function in potential living kidney donors (5), and it was the method used in the initial description of the model by Benoit et al. (11), although the external validation by Kulik et al. (12) used the MDRD formula to calculate the eGFR pre and after donation.

The risk of ESRD in living donors, although marginal, was evidenced in two studies in comparison with healthy controls (8, 9). As ESRD is a rare event, its surrogates have been pursued by several groups to improve living donor selection and donor safety. CKD, as defined by eGFR < 60 mL/min/1.73 m^2^, was associated with an increased risk of death, cardiovascular events, and hospitalization in a large, community-based population (10). In a registry-based cohort study of 71,468 living kidney donors, reported an independent association of living kidney donor eGFR at postoperative 6 months and subsequent ESRD. A 10 mL/min/1.73 m^2^ difference in early post-donation estimated glomerular filtration rate was significantly associated with a 28% higher risk of subsequent end-stage renal disease (17). However, no significant association has been found with the preoperative eGFR (17), and no marker could be identified in pre donation evaluation. One-year post-donation eGFR was assessed in this study, and it can be assumed as a surrogate of long-term renal function in the donor. We hypothesize that donors with lower eGFR 1 year after donation would benefit from increased surveillance and further preventive measures for renal health. Considering the global performance of this formula, we can go further and hypothesize that at pre-donation consultation, potential donors with predicted lower 1-year eGFR could be considered unfit to donate, after a global risk assessment, considering donor age and expected lifespan.

Benoit et al. (11) developed a model to estimate the donor’s 1-year post-donation eGFR. In this predictive model, Age and preoperative eGFR were shown to be independent predictors of 1-year postoperative renal function. Other donor characteristics like kidney size, gender, hypertension, obesity, dyslipidemia, and smoking were not found to influence the 1-year postoperative eGFR (11). In contrast, a recent retrospective study from Lam et al. (18) evaluated a Canadian cohort of living kidney donors and allowed a better understanding of kidney function over 5 years after living donor nephrectomy. In this study, changes in eGFR after donation varied by sex, percent decline in eGFR within the first 6 weeks after donation, and eGFR category at 1 year, but not by age category at donation, pre-donation hypertension, pre-donation eGFR category, socioeconomic status, or distance to transplant center (18). Be it as it may, the predictive model developed from the Toulouse-Rangueil cohort unquestionably showed a good correlation between predicted and observed donor eGFR 1-year after donation in 3 different centers (11–13). These results, along with the fact that donor age was found to be a strong predictor of CKD after LDKT, may defer the wish to extend, without fair criticism, the age limit of donors, which has been advocated to expand the pool (19, 20). A global risk assessment must always guide the clinical decision.

At the original cohort (11), 22.4% of donors had CKD at 1-year after donation, meeting KDIGO criteria of CKD (21). Kullik et al. (12), in the external validation in a German cohort, found a surprisingly higher incidence of CKD in their LKD cohort: 70.8%. A careful interpretation is needed as eGFR was calculated using the MDRD formula and not CKD-EPI. Additionally, the authors refer that at least 30% of all living donors preferred external follow-up appointments and were not included in the study. In our population, 25.5% of donors (85 out of 333) reached the definition of CKD, although none had ESRD at long-term follow-up. These donors represent a population that deserves more careful long-term surveillance. Further studies are necessary to evaluate the different trajectories of the long-term evolution of kidney function in these donors. It is recognized that some groups of living donors have a higher long-term risk of ESRD than others. Massie et al. (22) used data from the Scientific Registry of Transplant Recipients of 133,824 living kidney donors in the United States between 1978 and 2015 to construct a risk calculator that includes sex, Age, race, BMI, and first-degree biological relationship. Male sex, black race, older Age in the non-black race, greater body mass index, and first-degree biological relationship to the recipient were associated with increased risk of ESRD (22). Although the predicted 20-year risk of ESRD for the median donor was only 34 cases per 10,000 donors, 1% of donors had predicted risk exceeding 256 cases per 10,000 donors (22). Ibrahim et al. (23) used data from the University of Minnesota from 3,956 White kidney donors between 1963 and 2013. Their calculator estimates ESRD risk in White donors using Age, BMI, and systolic blood pressure at the time of donation (23). ESRD was associated with older age, higher BMI, and higher systolic blood pressure in the donation (23).

Most of our living donors were females (71%). Women are more likely than men to become living kidney donors (24, 25). In a recently published review of country-specific sex disparities in living kidney donation (26), Kurnikowski et al., described a population size-weighted donor distribution consisting of 35.9% men and 64.1% women. This data cannot be explained by a comprehensive reason (24). Biological and sociocultural aspects must be considered. Biological reasons usually described include the sex distributions of some potential biological risk factors for disease, including smoking, and a higher incidence of hypertension and ischemic heart disease that can preclude the acceptance of male candidates more often. Although women have a higher prevalence of chronic kidney disease than men, end-stage renal disease incidence is higher in men (24). Socio-cultural aspects are very significant in most cultures. It is expected that increased altruism from women, is derived from the women’s more traditional role as the caregiver in the family (25–27). The family expectations frequently remain on her to be a living donor, whether it remains on the man to keep working and support the entire family. This is still very common in Portuguese society nowadays, mainly in the rural and less favored communities. The predictive model performance did not differ when both sexes were considered separately, although the optimal cutoff for the prediction of CKD was slightly lower in women.

We must recognize the limitations associated with this study, beginning with its retrospective and observational design. Thirty-one donors were excluded from the study because 1-year serum creatinine was unavailable to calculate eGFR. Still, later creatinine values were available and were not different from the rest of the cohort. We assume it would not compromise the results of our validation cohort. All patients were Caucasians, but they were representative of the Portuguese population. Other races and ethnic origins are not represented. We used CKD-EPI to calculate eGFR and not an isotopic method. However, we must point out the unsuitability of the latter in clinical practice, as it is not recommended as a standard of care by current guidelines (4), and the model itself was developed using the CKD-EPI formula. Although we must be aware of the potential risk of analysis bias judgment of the original model, it should not preclude the results of this and the other external validation results.

The primary goal in assessing a living donor candidate must ensure minimal risk to the donor. Hence, the prediction of postoperative renal function is a critical point in their evaluation and, in our population, can be achieved with this tool. Furthermore, the required variables are low-cost and easily assessed, so its potential as a counseling tool is undeniable. We recall, however, that validation out of Europe is lacking and that further studies are necessary to validate prognostic models for longer-term prediction of donor kidney function.

## Conclusion

The formula developed in Toulouse-Rangueil was successfully validated in our cohort, a different European population than previously described. We must, anyway, emphasize that the optimal value of predicted eGFR was around 5 mL/min higher than the equality cutoff for CKD detection at 1 year. This model represents a simple and accurate tool that may be used to assist in the evaluation of potential donors, particularly in the current setting of increasing donor age, donors with minor comorbidities, or renal function close to the accepted threshold.

## Data Availability

The raw data supporting the conclusion of this article will be made available by the authors, without undue reservation.

## Abbreviations

AUC, area under the curve; BMI, body mass index; CI, confidence interval; CITL, calibration in the large; CKD, chronic kidney disease; CKD-EPI, chronic kidney disease epidemiology collaboration; ESRD, end-stage renal disease; eGFR, estimated glomerular filtration rate; GFR, glomerular filtration rate; LKD, living kidney donor; MDRD, modification of diet in renal disease; NPV, negative predictive value; PPV, positive predictive value; ROC, receiver operating characteristic; SD, standard deviation.

## References

[1] Gruessner RWG Gruessner AC. Solid-organ Transplants from Living Donors: Cumulative United States Experience on 140,156 Living Donor Transplants over 28 Years. Transpl Proc (2018) 50(10):3025–35. 10.1016/j.transproceed.2018.07.024 30577162

[2] Park YH Min SK Lee JN Lee HH Jung WK Lee JS Comparison of Survival Probabilities for Living-Unrelated versus Cadaveric Renal Transplant Recipients. Transpl Proc (2004) 36(7):2020–2. 10.1016/j.transproceed.2004.08.122 15518731

[3] Meier-Kriesche HU Kaplan B. Waiting Time on Dialysis as the Strongest Modifiable Risk Factor for Renal Transplant Outcomes: a Paired Donor Kidney Analysis. Transplantation (2002) 74(10):1377–81. 10.1097/00007890-200211270-00005 12451234

[4] Lentine KL Kasiske BL Levey AS Adams PL Alberu J Bakr MA Summary of Kidney Disease: Improving Global Outcomes (KDIGO) Clinical Practice Guideline on the Evaluation and Care of Living Kidney Donors. Transplantation (2017) 101(8):1783–92. 10.1097/TP.0000000000001770 28737659 PMC5542788

[5] Andrews PA Burnapp L. British Transplantation Society/Renal Association UK Guidelines for Living Donor Kidney Transplantation 2018: Summary of Updated Guidance. Transplantation (2018) 102(7):e307. 10.1097/TP.0000000000002253 29688993 PMC7228639

[6] Reese PP Caplan AL Kesselheim AS Bloom RD. Creating a Medical, Ethical, and Legal Framework for Complex Living Kidney Donors. Clin J Am Soc Nephrol (2006) 1(6):1148–53. 10.2215/CJN.02180606 17699340

[7] Reese PP Boudville N Garg AX. Living Kidney Donation: Outcomes, Ethics, and Uncertainty. Lancet (2015) 385(9981):2003–13. 10.1016/S0140-6736(14)62484-3 26090646

[8] Muzaale AD Massie AB Wang MC Montgomery RA McBride MA Wainright JL Risk of End-Stage Renal Disease Following Live Kidney Donation. JAMA (2014) 311(6):579–86. 10.1001/jama.2013.285141 24519297 PMC4411956

[9] Mjoen G Hallan S Hartmann A Foss A Midtvedt K Oyen O Long-term Risks for Kidney Donors. Kidney Int (2014) 86(1):162–7. 10.1038/ki.2013.460 24284516

[10] Go AS Chertow GM Fan D McCulloch CE Hsu CY. Chronic Kidney Disease and the Risks of Death, Cardiovascular Events, and Hospitalization. N Engl J Med (2004) 351(13):1296–305. 10.1056/NEJMoa041031 15385656

[11] Benoit T Game X Roumiguie M Sallusto F Doumerc N Beauval JB Predictive Model of 1-year Postoperative Renal Function after Living Donor Nephrectomy. Int Urol Nephrol (2017) 49(5):793–801. 10.1007/s11255-017-1559-1 28251483

[12] Kulik U Gwiasda J Oldhafer F Kaltenborn A Arelin V Gueler F External Validation of a Proposed Prognostic Model for the Prediction of 1-year Postoperative eGFR after Living Donor Nephrectomy. Int Urol Nephrol (2017) 49(11):1937–40. 10.1007/s11255-017-1683-y 28828572

[13] Benoit T Prudhomme T Adypagavane A Malavaud B Soulie M Game X External Validation of a Predictive Model to Estimate Renal Function after Living Donor Nephrectomy. Transplantation (2021) 105(11):2445–50. 10.1097/TP.0000000000003643 33496555

[14] Collins GS Reitsma JB Altman DG Moons KGM. Transparent Reporting of a Multivariable Prediction Model for Individual Prognosis or Diagnosis (TRIPOD). Ann Intern Med (2015) 162(10):735–6. 10.7326/L15-5093-2 25984857

[15] Levey AS Stevens LA Schmid CH Zhang YL Castro AF 3rd Feldman HI A New Equation to Estimate Glomerular Filtration Rate. Ann Intern Med (2009) 150(9):604–12. 10.7326/0003-4819-150-9-200905050-00006 19414839 PMC2763564

[16] Stevens LA Schmid CH Greene T Zhang YL Beck GJ Froissart M Comparative Performance of the CKD Epidemiology Collaboration (CKD-EPI) and the Modification of Diet in Renal Disease (MDRD) Study Equations for Estimating GFR Levels above 60 mL/min/1.73 M2. Am J Kidney Dis (2010) 56(3):486–95. 10.1053/j.ajkd.2010.03.026 20557989 PMC2926290

[17] Massie AB Holscher CM Henderson ML Fahmy LM Thomas AG Al Ammary F Association of Early Postdonation Renal Function with Subsequent Risk of End-Stage Renal Disease in Living Kidney Donors. JAMA Surg (2020) 155(3):e195472. 10.1001/jamasurg.2019.5472 31968070 PMC6990748

[18] Lam NN Lloyd A Lentine KL Quinn RR Ravani P Hemmelgarn BR Changes in Kidney Function Follow Living Donor Nephrectomy. Kidney Int (2020) 98(1):176–86. 10.1016/j.kint.2020.03.034 32571482

[19] Serrano OK Yadav K Bangdiwala A Vock DM Dunn TB Finger EB Age Alone Is Not a Contraindication to Kidney Donation: Outcomes of Donor Nephrectomy in the Elderly. Clin Transpl (2018) 32(8):e13287. 10.1111/ctr.13287 29923234

[20] Klop KW Dols LFC Weimar W Dooper IM Ijzermans JNM Kok NFM. Quality of Life of Elderly Live Kidney Donors. Transplantation (2013) 96(7):644–8. 10.1097/TP.0b013e31829e6d9b 23860088

[21] Levin A Stevens PE. Summary of KDIGO 2012 CKD Guideline: behind the Scenes, Need for Guidance, and a Framework for Moving Forward. Kidney Int (2014) 85(1):49–61. 10.1038/ki.2013.444 24284513

[22] Massie AB Muzaale AD Luo X Chow EKH Locke JE Nguyen AQ Quantifying Postdonation Risk of ESRD in Living Kidney Donors. J Am Soc Nephrol (2017) 28(9):2749–55. 10.1681/ASN.2016101084 28450534 PMC5576930

[23] Ibrahim HN Foley RN Reule SA Spong R Kukla A Issa N Renal Function Profile in White Kidney Donors: The First 4 Decades. J Am Soc Nephrol (2016) 27(9):2885–93. 10.1681/ASN.2015091018 26888476 PMC5004661

[24] Katz-Greenberg G Shah S. Sex and Gender Differences in Kidney Transplantation. Semin Nephrol (2022) 42(2):219–29. 10.1016/j.semnephrol.2022.04.011 35718368 PMC10065984

[25] Carrero JJ Hecking M Chesnaye NC Jager KJ. Sex and Gender Disparities in the Epidemiology and Outcomes of Chronic Kidney Disease. Nat Rev Nephrol (2018) 14(3):151–64. 10.1038/nrneph.2017.181 29355169

[26] Kurnikowski A Krenn S Lewandowski MJ Schwaiger E Tong A Jager KJ Country-specific Sex Disparities in Living Kidney Donation. Nephrol Dial Transpl (2022) 37(3):595–8. 10.1093/ndt/gfab305 PMC8875465 34669961

[27] Brar A Markell M. Impact of Gender and Gender Disparities in Patients with Kidney Disease. Curr Opin Nephrol Hypertens (2019) 28(2):178–82. 10.1097/MNH.0000000000000482 30652978

